# Diagnostic accuracy of molecular methods for detecting markers of antimalarial drug resistance in clinical samples of *Plasmodium falciparum*: protocol for an update to a systematic review and meta-analysis

**DOI:** 10.1186/s13643-018-0891-6

**Published:** 2018-12-05

**Authors:** Rebekah Burrow, Thomas R. Fanshawe, Georgina S. Humphreys

**Affiliations:** 1 Department for Continuing Education, University of Oxford, Oxford, UK; 2 Nuffield Department of Primary Care Health Sciences, University of Oxford, Oxford, UK; 3 Green Templeton College, University of Oxford, Oxford, UK

**Keywords:** Malaria, Antimalarial, Resistance, Diagnostic accuracy, Molecular, Systematic review, Protocol

## Abstract

**Background:**

Each year, infection with *Plasmodium* causes millions of clinical cases of malaria and hundreds of thousands of deaths. Resistance to different antimalarial medications continues to develop and spread, threatening effective prophylaxis and treatment. Surveillance of resistance is required to inform health policy and preserve effective antimalarial drugs; molecular methods can be used to surveil likely parasite resistances. However, there is no consensus on the most accurate molecular methods, and large variation exists in practice. The objective of this update to this systematic review is to improve and update identification of the sensitivity and specificity of each molecular method for detecting selected antimalarial drug resistance markers.

**Methods:**

We will include diagnostic accuracy studies that compare at least two of any molecular methods to examine blood samples from patients diagnosed with, or suspected of having malaria, to detect at least one selected marker of antimalarial drug resistance. We will search PubMed, EMBASE, BIOSIS, and Web of Science from 2000 to present. Two reviewers will independently screen all results, extract data, consider applicability, and evaluate the methodological quality of included studies using QUADAS-2. We will carry out a meta-analysis and use statistical methods to compare results from homogenous studies. We will use narrative to synthesise and compare results of heterogeneous studies.

**Discussion:**

This review will help to identify sub-optimal molecular methods for antimalarial marker detection which may be discontinued and identify more sensitive and specific methods which may be adopted. More sensitive and specific detection of drug resistance can be used to improve the breadth and accuracy of surveillance. This would enable the identification of previously undiscovered areas of antimalarial resistances and susceptibilities, improve the precision of estimates of the prevalence of resistances, and improve our ability to detect smaller changes in these patterns. Higher-quality evidence generated by more accurate and detailed surveillance can be used to inform guidelines on the use of antimalarial drugs, leading to better outcomes for more patients.

**Systematic review registration:**

This systematic review protocol was registered with PROSPERO on 22 November 2017 (registration number CRD42017082101).

**Electronic supplementary material:**

The online version of this article (10.1186/s13643-018-0891-6) contains supplementary material, which is available to authorized users.

## Introduction

Infection with *Plasmodium* caused approximately 216 million clinical cases of malaria and 445,000 deaths in 2016 [1]. However, malaria can be prevented and treated effectively [2, 3]. However, some *Plasmodium falciparum* (*P. falciparum*) parasites are resistant, or less susceptible, to some antimalarial chemotherapies [4–13]. The emergence and spread of drug-resistant parasites are a great impediment to achieving control of malaria and eventual elimination.

While worldwide surveillance of artemisinin resistance has been prioritised, surveillance of resistances to other prophylactic and curative antimalarials is also essential [14–17]. Continuous global surveillance of therapeutic efficacy and drug resistance is enabling the detection of epidemiological patterns of parasite resistances to antimalarial drugs in order to inform malaria prevention and treatment policies [18, 19].

Historically, parasite resistances have been determined using clinical trials or observational studies. In addition, for a limited number of clinical samples, therapeutic efficacy can be tested through ex vivo and in vitro culture methods. Molecular methods may offer many advantages over both of these: reduced patient burden, reduced number of staff, faster results, and the opportunity to test possible resistances to many antimalarial drugs [14, 20–22]. However, the correlation between the genotypes of markers and the phenotypes of resistances is mixed in strength for different molecular markers; the clinical relevance of these molecular markers varies [23, 24].

There is a lack of a standardised protocol for detecting resistance via molecular markers, with variation in sample type, collection, storage, DNA extraction, marker detection, and analysis of results, all of which may also affect the sensitivity and specificity of methods [14, 25].

It seems likely that there is a variation in the accuracy of different molecular methods for detecting antimalarial drug resistance. For example, for similar patients, Sarmah et al. [26] determined *pfdhfr* N51I, C59R, and S108N for 75/75 samples, while Mishra et al. [27] were successful for 232/311 samples.

An updated systematic review will identify the molecular methods in use and the sensitivity and specificity of each method for detecting each drug resistance marker. Any methods found to be sub-optimal in a particular setting could be discontinued from use and replaced by the more sensitive and specific methods. Use of more sensitive and specific methods could require fewer resources to generate an equal amount of data. It could result in fewer gaps in surveillance and increased accuracy of surveillance. Improved surveillance could identify previously undiscovered pockets of antimalarial resistances and susceptibilities, improve the accuracy and precision of estimates of known resistance, and improve our ability to detect smaller changes in these patterns.

Improved guidelines on the use of antimalarial drugs, based on this more complete, accurate, and precise information, would likely lead to better outcomes for more patients.

One review on the subject has been carried out; the sensitivities and specificities of many methods were determined in different settings. However, the confidence intervals were large, and the risks of bias were unclear for many studies. In addition, this review was carried out by a single reviewer; an update and extension to the first review is required to improve the methodological quality of the review and to include additional studies [28].

## Objectives

The objectives of this review are to:

- Obtain per study estimates of the sensitivity and specificity of each molecular method for determining the genotype of each drug resistance marker.
- Obtain summary estimates of the sensitivity and specificity of each molecular method for determining the genotype of each drug resistance marker.
- Use statistical methods to compare estimates of sensitivity and specificity of different methods to identify the most sensitive and specific methods.
- Narratively synthesise and compare estimates of sensitivity and specificity to identify the most sensitive and specific methods when statistical synthesis and comparison are not appropriate.

## Methods

This protocol was designed and prepared following guidance from PRISMA, PRISMA-Abstracts, PRISMA-P, and PRISMA-DTA guidelines [29–35].

### Eligibility criteria

We will select studies according to the criteria below:

- We will include only diagnostic accuracy studies. We will include case-control studies in the review but may exclude them from the meta-analysis.
- We will include studies examining any number of blood samples, taken from patients or asymptomatic patients when there is a suspicion that they may be carrying *P. falciparum* parasites. We will exclude data generated using artificial infections, lab-adapted *Plasmodium* strains, and ‘spiked’ samples.
- We will include as index and reference tests any molecular methods for determining the presence or the genotype of at least one molecular marker associated with antimalarial drug resistance. We will include all studies comparing at least two methods. There is no gold standard test and no exhaustive list of methods. By including all methods, we aim to include evidence applicable to all surveillance settings. This was a successful technique for the first iteration of this review.
- The target condition is the genotype, haplotype, or copy number of a molecular marker of antimalarial drug resistance; the genes containing these markers are listed in Table 1. We will include studies that examine at least one of these genes.
- We will include studies for which sufficient data to construct a 2 × 2 table of test accuracy is published or can be obtained from authors. When results to construct a 2 × 2 table are not recovered, studies will be listed but will not be included in the quantitative or narrative syntheses.
- We will include studies from any setting, regardless of the prevalence of *Plasmodium* or presence of different *Plasmodium* species.
- We will not restrict studies by language of publication.
- We will include only studies for which there is published report.

**Table 1:** Associations of mutations in *P. falciparum* with antimalarial resistance

Gene	Associated with susceptibility or resistance to
*Chloroquine resistance transporter* (*pfcrt*)	Amodiaquine, chloroquine, lumefantrine, quinine
*Cytochrome b* (*pfcytb*)	Atovaquone
*Dihydrofolate reductase* (*pfdhfr*)	Cycloguanil, proguanil, pyrimethamine
*Dihydropteroate synthase* (*pfdhps*)	Sulphadoxine
*Kelch 13* (*pfk13*)	Artemisinins
*Multi-drug resistance 1* (*pfmdr1*)	Amodiaquine, artemisinins, chloroquine, halofantrine, lumefantrine, mefloquine, piperaquine, quinine
*Plasmepsin 2* (*pfpm2*)	Piperaquine
*Plasmepsin 3* (*pfpm3*)	Piperaquine

### Information sources

We will search PubMed, EMBASE (Ovid), BIOSIS (Web of Knowledge), and Web of Science Core Collection (Web of Knowledge) from 2000 to present. We will not search for studies published before 2000 as none were found in the comprehensive search from 1983 undertaken in the first iteration of this review. We will check reference lists of eligible studies identified through the search. We will circulate a list of the eligible reports to authors of included reports and to experts in malaria diagnostics and drug resistance that we have identified. We will make an initial search and a second search later in the review to check for new studies. We will utilise literature search strategies using medical subject headings (MeSH) and terms related to malaria, drug resistance, genes of interest, and molecular methods. No filters will be used. Piloted search terms for all databases are included in Additional file 1.

### Data management

We will upload literature search results to EndNote 8.2 software and Covidence.

### Selection process

We will assess titles or titles and abstracts of search results using a piloted screening checklist based on our inclusion and exclusion criteria; the screening checklist is available in Additional file 2. We will obtain the full text of reports for any potentially eligible studies or when there is any uncertainty. We will use the same screening form to assess full-text reports.

We will use report and study details to identify multiple reports of the same study or samples; we will compare any potential duplicate reports in full. At any stage of the review, if we find multiple reports of the same study, all but one report will be excluded. A second reviewer will independently screen the search results. A third reviewer will resolve disagreements. We will record at least one reason that reports or studies are excluded. Review authors will not be blind to the journal titles, study authors, or institution.

### Data collection process

We will extract data from all eligible studies using piloted standardised data extraction forms; the data extraction forms are available in Additional file 3. Two reviewers will independently extract all data. Disagreements will be resolved by a third reviewer. When necessary, we will seek from authors, using the results data extraction form in Additional file 4, unpublished data to construct 2 × 2 tables of test accuracy.

### Data items

We will extract report and publication details. We will extract study identifiers and characteristics including design, location and time, patient and sample characteristics, molecular methods used and markers examined, flow and timing of patients, and samples through testing. We will extract details on potential sources of bias for assessment using the QUADAS-2 (Quality Assessment of Diagnostic Accuracy Studies) checklist and other items recommended by STARD (Standards for Reporting of Diagnostic Accuracy) [36, 37]. We will extract all results relating to test accuracy. A full list of data items is available in Additional file 3.

Sensitivity and specificity will be calculated from any test accuracy outcomes provided. If data cannot be obtained in a numeric format, it will be translated from graphically presented data using im2graph 1.21.

### Outcomes and prioritisation

The primary outcome will be:

- The sensitivity and specificity from each study, of each molecular method for determining the genotype of any drug resistance markers in any gene listed in Table 1.

### Risk of bias in individual studies

We will assess the risk of bias using the piloted adapted QUADAS-2 tool in Additional file 4. For each domain, two reviewers will independently make an assessment of the risk of bias from the extracted information and rate the risk of bias as “low”, “high”, or “unclear”. If insufficient data is available in reports or obtained as planned, the risk of bias will be rated as “unclear”. Disagreements will be resolved by a third reviewer. In addition, we will consider other sources of bias. We will create a graphical representation of risk of bias within included studies using Covidence. We will describe how the results of the assessment contribute to the overall findings of the review.

### Data synthesis

We will perform analyses using Stata 15 and display key characteristics in tables. For each study, we will determine sensitivity and specificity with 95% confidence intervals (CIs). Where haplotypes are reported, they will be synthesised independently to genotypes. It is anticipated that thresholds of test positivity and details of multiple test readers will not be available in published reports, as found in the initial iteration of this review. Indeterminate test results will be used to construct 3 × 3 tables of test accuracy and included in calculations of sensitivity and specificity. Index and reference tests will be grouped by exact methods.

### Meta-analysis

We will follow the methods recommended by the Cochrane Collaboration Handbook for Diagnostic Test Accuracy Reviews [33]. Where we judge that studies are sufficiently similar in design, setting, patient and sample characteristics, and test conduct, we will plot their sensitivity and specificity estimates in ROC space and conduct meta-analyses using a bivariate random-effects model to generate and compare summary estimates of sensitivity and specificity.

If we carry out a meta-analysis, we will use a meta-regression to examine heterogeneity in relation to study setting, patient and sample characteristics, and test conduct. We will carry out a sensitivity analysis to examine any assumptions that combining studies with different study settings, patient and sample characteristics, and test conduct was justified. We will use hierarchical logistic regression to compare estimates of sensitivity and specificity for different tests. We will calculate correlation coefficients to test relatedness of sensitivity and specificity.

Where studies are heterogeneous in design, setting, patient and sample characteristics, or test conduct, we will not carry out a meta-analysis and will synthesise the data through narrative, following guidance from Popay et al. [38].

### Publication bias

We will not carry out a formal assessment of publication bias of studies included in this review; techniques such as funnel plots and regression tests have not been found to be useful for identifying publication bias of diagnostic test accuracy studies [39].

### Confidence in cumulative estimate

We will assess the quality of evidence using Grading of Recommendations Assessment, Development and Evaluation (GRADE) methodology [40]. We will consider additional domains where appropriate. We will designate quality as high (further research is very unlikely to change our confidence in the estimate of effect), moderate (further research is likely to have an important impact on our confidence in the estimate of effect and may change the estimate), low (further research is very likely to have an important impact on our confidence in the estimate of effect and is likely to change the estimate), or very low (very uncertain about the estimate of effect).

## Discussion

This updated review will help to identify sub-optimal molecular methods for antimalarial marker detection which may be discontinued and identify more sensitive and specific methods which may be adopted. More sensitive and specific detection of drug resistance can be used to improve the breadth and accuracy of surveillance. This would enable the identification of previously undiscovered areas of antimalarial resistances and susceptibilities, improve the precision of estimates of the prevalence of resistances, and improve our ability to detect smaller changes in these patterns. Higher-quality evidence generated by more accurate and detailed surveillance can be used to inform guidelines on the use of antimalarial drugs, leading to better outcomes for more patients.

## Additional files

Additional file 1: Piloted search terms. (DOCX 15 kb)

Additional file 2: Piloted screening checklist. (DOCX 13 kb)

Additional file 3: Piloted data extraction forms. (DOCX 15 kb)

Additional file 4: Piloted modified QUADAS-2 risk of bias tool. (DOCX 13 kb)

## Abbreviations

pfcrt: *Plasmodium falciparum* chloroquine resistance transporter
pfcytb: *Plasmodium falciparum* cytochrome b
pfdhfr: *Plasmodium falciparum* dihydrofolate reductase
pfdhps: *Plasmodium falciparum* dihydropteroate synthase
pfk13: *Plasmodium falciparum* kelch 13
pfmdr1: *Plasmodium falciparum* multi-drug resistance transporter 1
pfpm2: *Plasmodium falciparum* plasmepsin 2
pfpm3: *Plasmodium falciparum* plasmepsin 3
