# Supplementary material for: Diagnostic accuracy of molecular methods for detecting markers of antimalarial drug resistance in clinical samples of Plasmodium falciparum: protocol for an update to a systematic review and meta-analysis
Source: Syst Rev. 2018 Dec 5;7:221. doi: 10.1186/s13643-018-0891-6 (PMC6280367; doi:10.1186/s13643-018-0891-6)
Supplement: Supplementary file 1 — Piloted search terms. (DOCX 15 kb) [file 13643_2018_891_MOESM1_ESM.docx]

Additional File 1 – Piloted search terms

1. PubMed

(("plasmodium falciparum") AND ("resist*" OR "suscept*" OR "sensit*") AND ("pfcrt" OR "crt" OR "chloroquine resistance transporter" OR "pfcytb" OR "cytb" OR "pfcyt b" OR "cyt b" OR "cytochrome b" OR "cytochromes b"[MeSH Terms] OR "pfdhfr" OR "dhfr" OR "dihydrofolate reductase" OR "tetrahydrofolate dehydrogenase"[MeSH Terms] OR "pfdhps" OR "dhps" OR "dihydropteroate synthase" OR "pfk13" OR "k13" OR "pfk 13" OR "k 13" OR "kelch13" OR "kelch 13" OR "pfmdr1" OR "mdr1" OR "pfmdr 1" OR "mdr 1" OR "multi-drug resistance 1" OR "multidrug resistance 1" OR "multidrug resistance1" OR "pfmrp" OR "plasmepsin 2" OR "plasmepsin2" OR ("plasmepsin" AND "3")))

1. EMBASE

(("plasmodium falciparum") AND ("resist*" OR "suscept*" OR "sensit*") AND ("pfcrt" OR "crt" OR "chloroquine resistance transporter" OR "pfcytb" OR "cytb" OR "pfcyt b" OR "cyt b" OR "cytochrome b" OR "pfdhfr" OR "dhfr" OR "dihydrofolate reductase" OR "pfdhps" OR "dhps" OR "dihydropteroate synthase" OR "pfk13" OR "k13" OR "pfk 13" OR "k 13" OR "kelch13" OR "kelch 13" OR "pfmdr1" OR "mdr1" OR "pfmdr 1" OR "mdr 1" OR "multi-drug resistance 1" OR "multidrug resistance 1" OR "multidrug resistance1" OR "pfmrp" OR "plasmepsin 2" OR "plasmepsin2" OR ("plasmepsin" AND "3")))

1. BIOSIS

(TS=("plasmodium falciparum") AND TS=("resist*" OR "suscept*" OR "sensit*") AND TS=("pfcrt" OR "crt" OR "chloroquine resistance transporter" OR "pfcytb" OR "cytb" OR "pfcyt b" OR "cyt b" OR "cytochrome b" OR "pfdhfr" OR "dhfr" OR "dihydrofolate reductase" OR "pfdhps" OR "dhps" OR "dihydropteroate synthase" OR "pfk13" OR "k13" OR "pfk 13" OR "k 13" OR "kelch13" OR "kelch 13" OR "pfmdr1" OR "mdr1" OR "pfmdr 1" OR "mdr 1" OR "multi-drug resistance 1" OR "multidrug resistance 1" OR "multidrug resistance1" OR "pfmrp" OR "plasmepsin 2" OR "plasmepsin2" OR ("plasmepsin" AND "3")))

1. Web of Science Core Collection

(TS=("plasmodium falciparum") AND TS=("resist*" OR "suscept*" OR "sensit*") AND TS=("pfcrt" OR "crt" OR "chloroquine resistance transporter" OR "pfcytb" OR "cytb" OR "pfcyt b" OR "cyt b" OR "cytochrome b" OR "pfdhfr" OR "dhfr" OR "dihydrofolate reductase" OR "pfdhps" OR "dhps" OR "dihydropteroate synthase" OR "pfk13" OR "k13" OR "pfk 13" OR "k 13" OR "kelch13" OR "kelch 13" OR "pfmdr1" OR "mdr1" OR "pfmdr 1" OR "mdr 1" OR "multi-drug resistance 1" OR "multidrug resistance 1" OR "multidrug resistance1" OR "pfmrp" OR "plasmepsin 2" OR "plasmepsin2" OR ("plasmepsin" AND "3")))
