# Supplementary material for: Diagnostic accuracy of molecular methods for detecting markers of antimalarial drug resistance in clinical samples of Plasmodium falciparum: protocol for an update to a systematic review and meta-analysis
Source: Syst Rev. 2018 Dec 5;7:221. doi: 10.1186/s13643-018-0891-6 (PMC6280367; doi:10.1186/s13643-018-0891-6)
Supplement: Supplementary file 2 — Piloted screening checklist. (DOCX 13 kb) [file 13643_2018_891_MOESM2_ESM.docx]

Additional File 2 – Piloted screening checklist

| Inclusion Criteria | 1. Diagnostic accuracy study design, including case-control |
| --- | --- |
|  | 1. Studies examining clinical blood samples taken from patients or asymptomatic patients when there is a knowledge or suspicion that they may be carrying *Plasmodium* parasites |
|  | 1. Studies examining at least one antimalarial resistance marker in at least one of the genes listed in Table 1. |
|  | 1. Studies comparing any two or more molecular methods for detecting an antimalarial resistance marker in a gene in Table 1. |
|  | 1. Studies publishing or providing sufficient data to construct a 2x2 table of test accuracy |
| Exclusion criteria | 1. Data generated using artificial infections, lab-adapted *Plasmodium* strains and ‘spiked’ samples. |
|  | 1. Studies with no published report |
