# Supplementary material for: Diagnostic accuracy of molecular methods for detecting markers of antimalarial drug resistance in clinical samples of Plasmodium falciparum: protocol for an update to a systematic review and meta-analysis
Source: Syst Rev. 2018 Dec 5;7:221. doi: 10.1186/s13643-018-0891-6 (PMC6280367; doi:10.1186/s13643-018-0891-6)
Supplement: Supplementary file 3 — Piloted data extraction forms. (DOCX 15 kb) [file 13643_2018_891_MOESM3_ESM.docx]

Additional File 3 – Piloted data extraction forms

1. Main details (one per study)

| Reviewer | Reviewer 1 name and date |
| --- | --- |
|  | Reviewer 2 name and date |
|  | Request more information? |
| Report | Published report title |
|  | First author name |
|  | Date report published |
| Dates | *Plasmodium* infection |
|  | Patient recruitment |
|  | Sample collection |
|  | Sample testing analysis |
| Study | Study site location |
|  | Study site setting |
|  | Laboratory location |
|  | Laboratory type |
|  | Design |
| Patients | Age, sex |
|  | Coinfections |
|  | Eligibility criteria, symptoms |
|  | Screening / active case finding |
|  | Method of diagnosis |
|  | Method of sampling |
|  | Number of patients |
| Samples | Country of *Plasmodium* infection |
|  | Source of blood |
|  | Method of parasite count |
|  | Parasitaemia |
|  | Blood storage |
|  | Method of DNA extraction |
|  | DNA storage |
|  | Method of speciation |
|  | Species |
|  | Method of multiplicity |
|  | Multiplicity of infection |
|  | Method of sampling |
|  | Number of samples |
| Detection of | Genes |
|  | Markers |
|  | Alleles |
| Reference standard(s) | Name |
| Index test(s) | Name |
| Blinding | Of whom, how |
| Interval | Between sampling |
|  | Between tests |
| Potential COI | List of funding, affiliations, COIs |

1. Flow of patients and samples (one per study)

| Patients considered for inclusion |
| --- |
| Patients selected for wider study |
| Patients selected for diagnostic accuracy study |
| Samples selected for diagnostic accuracy study |
| Samples included in the analysis |
| Samples analysed by each test |

1. Details of molecular methods (One per method)

| Molecular method | Index/Reference |
| --- | --- |
|  | Compared to |
|  | Conducted by |
|  | Conduct |
|  | Analysis |
|  | Interpretation |

1. 3x3 results table (One per comparison of methods for each marker)

| gene | position of marker | Reference standard | | |
| --- | --- | --- | --- | --- |
|  |  | Mutation | Wildtype | Undetermined |
| Index test | Mutation |  |  |  |
|  | Wildtype |  |  |  |
|  | Undetermined |  |  |  |
