# Supplementary material for: Diagnostic accuracy of molecular methods for detecting markers of antimalarial drug resistance in clinical samples of Plasmodium falciparum: protocol for an update to a systematic review and meta-analysis
Source: Syst Rev. 2018 Dec 5;7:221. doi: 10.1186/s13643-018-0891-6 (PMC6280367; doi:10.1186/s13643-018-0891-6)
Supplement: Supplementary file 4 — Piloted modified QUADAS-2 risk of bias tool. (DOCX 13 kb) [file 13643_2018_891_MOESM4_ESM.docx]

Additional File 4 – Piloted modified QUADAS-2 risk of bias tool

| DOMAIN 1:  Patient Selection | Item |
| --- | --- |
|  | Was a consecutive or random sample of patients (or blood samples) enrolled? |
|  | Was a case-control design avoided? Was a range of parasitaemias used? |
|  | Did the study avoid inappropriate exclusions? |
|  | Risk of bias: Could the selection of patients have introduced bias? |
| DOMAIN 2:  Index Test | Applicability: Are there concerns that the included patients and setting do not match the review question? |
|  | Were the index test results interpreted without knowledge of the results of the reference standard (if tests were subjective)? |
|  | If a threshold was used, was it pre-specified and reasonable (e.g.: qPCR)? |
|  | Risk of Bias: Could the conduct or interpretation of the index test have introduced bias? |
| DOMAIN 3:  Reference Standard | Applicability: Are there concerns that the index test, its conduct, or interpretation differ from the review question? |
|  | Is the reference standard likely to correctly classify the target condition? |
|  | Were the reference standard results interpreted without knowledge of the results of the index tests? |
|  | If a threshold was used, was it pre-specified and reasonable (e.g.: qPCR)? |
|  | Risk of Bias: Could the reference standard, its conduct, or its interpretation have introduced bias? |
| DOMAIN 4:  Flow and Timing | Applicability: Are there concerns that the target condition as defined by the reference standard does not match the question? |
|  | Was there an appropriate interval between index test and reference standard (between sample taken)? |
|  | Did all patients receive the same reference standard? |
|  | Were all patients included in the analysis? |
|  | Risk of Bias: Could the patient flow have introduced bias? |
